# Sex-dominant differences in resting-state EEG microstates among young adults at risk of smartphone addiction

**DOI:** 10.3389/fnhum.2026.1714498

**Published:** 2026-04-27

**Authors:** Jun Chen, Yingying Zeng, Hui Liu, Danfeng Yu, Bo Yang, Qin Liu

**Affiliations:** 1 Department of Cardiopulmonary Rehabilitation, Taihe Hospital, Hubei University of Medicine, Shiyan, Hubei, China; 2 Department of Cardiology, Taihe Hospital, Hubei University of Medicine, Shiyan, Hubei, China; 3 Department of General Practice, Taihe Hospital, Hubei University of Medicine, Shiyan, Hubei, China; 4 Department of Neurocritical Care, Taihe Hospital, Hubei University of Medicine, Shiyan, Hubei, China; 5 Department of Radiology, Taihe Hospital, Hubei University of Medicine, Shiyan, Hubei, China

**Keywords:** EEG microstates, impulsivity, resting state, sex differences, smartphone addiction

## Abstract

**Background:**

Smartphone addiction (SA) is increasing, but sex-sensitive neurophysiological markers for assessment and follow-up remain limited.

**Methods:**

In a balanced 2 × 2 design (sex × group; *N* = 120), young adults had resting-state EEG. Microstates A–D were extracted from GFP peaks using polarity-insensitive k-means (*k* = 4). Individual metrics (mean duration, occurrence, coverage, GEV) were compared using two-way ANOVA with FDR control (*q* = 0.05).

**Results:**

Sex effects outperformed group effects. Females had higher microstate C metrics—duration: *F*(1, 116) = 12.84, *p* = 0.001, ηp^2^ = 0.100; coverage: *F*(1, 116) = 14.21, *p* < 0.001, ηp^2^ = 0.109; occurrence: *F*(1, 116) = 9.02, *p* = 0.003, ηp^2^ = 0.072, while males had higher microstate D metrics—coverage: *F*(1, 116) = 7.01, *p* = 0.009, ηp^2^ = 0.057; occurrence: *F* (1, 116) = 16.82, *p* < 0.001, ηp^2^ = 0.127; duration did not differ (*p* > 0.05). These patterns remained FDR-significant at *q* = 0.05.

**Conclusion:**

Resting EEG microstates reveal sex-dominant dynamics associated with salience (C) and dorsal attention (D) processes in SA with high impulsivity, indicating the need for sex-sensitive screening and follow-up in addition to questionnaires.

## Highlights

- A balanced 2 × 2 study (sex × group; *N* = 120) examined resting-state EEG microstates (A–D).
- Sex-dominant effects: C↑ in females (duration, coverage, and occurrence); D↑ in males (coverage, occurrence).
- D-duration was not significant; A/B showed no consistent differences.
- The findings are consistent with salience (C) vs. dorsal-attention (D) network tendencies.
- Exploratory connections between impulsivity, daily use, and IGT indicate sex-sensitive risk pathways.

## Introduction

As of June 2024, China had approximately 1.1 billion mobile internet users, with students being the majority (CNNIC, 2024). *Smartphone addiction, defined* as a loss of control over phone usage accompanied by compulsive, hard-to-curb engagement, has been associated with broad physiological, psychological, and social harms (Michel and Koenig, 2018; Khanna et al., 2015). Headache, insomnia, and tinnitus (Kwon et al., 2013), structural changes in the brain (Dieter et al., 2017), cognitive impairment, irritability, or depressed mood (Gao et al., 2018), reduced learning motivation (Soyemi et al., 2015; Jacobsen and Forste, 2011), interpersonal difficulties (Hao et al., 2019), and psychiatric disorders (Kuss et al., 2014) are among the reported side effects. Converging evidence suggest a negative associations between smartphone addiction and working memory (Billieux et al., 2008), executive function (Lepp et al., 2014), and response inhibition (Takao et al., 2009). Despite these well-documented adverse outcomes, it is unclear whether individuals with smartphone addiction and high impulsivity show sex-specific patterns across these domains—a question that has drawn increasing attention from researchers but lacks definitive evidence.

Smartphone addiction is classified as a behavioral (non-substance) addiction, which is more prevalent and widespread than substance addictions in everyday life. Behavioral addictions are driven by learned, cognitively mediated patterns of reinforcement rather than direct pharmacological action, whereas substance addictions are caused by dependency on exogenous drug use. Despite the differences in precipitants, the two classes have clinical features, such as decreased cognitive and motivational control, increased craving and cue reactivity to addiction-related stimuli, a preference for reward over risk avoidance, and impaired inhibitory control (He et al., 2017).

However, at the neural level, the overlap vs. specificity of mechanisms remains incompletely resolved. Evidence indicates that substance and behavioral addictions have partially overlapping activations in the striatum and prefrontal cortex, as well as divergent patterns: substance addictions often show aberrant activation within inhibitory-control circuits and reward-processing loops, whereas behavioral addictions more typically exhibit hyperactivity concentrated in reward-processing pathways (Luo et al., 2011; Wang et al., 2021). In this context, investigating sex differences in smartphone addiction provides an important vantage point for identifying common and distinct neurobiological mechanisms across substance and behavioral addictions.

Prior research has shown that smartphone addiction involves emotion processing and recognition (Chen et al., 2017), cognitive processing (Liu et al., 2017), and the reward system; nevertheless, the majority of data to far comes from fMRI, event-related potentials (ERPs), electrodermal (skin-conductance) tests, and behavioral paradigms. In contrast, EEG microstate analysis is a newer, high-temporal-resolution approach for tracking large-scale functional brain states evolve over time. It can characterize condition-dependent dynamics during cognitive processing and shed light on the generation and evolution of neural activity, with applications across brain science, brain disorders, psychology, and biomedicine.

Recent studies further indicates that both substance and behavioral addictions alter neurotransmitter signaling, resulting to downstream structural and functional brain changes (Peng et al., 2025; Horvath et al., 2020; Hill et al., 2023). Addiction mechanisms may also differ by sex (De-Sola Gutiérrez et al., 2016; Pan et al., 2013), with males and females exhibiting different EEG activity profiles and cognitive features (Tomescu et al., 2018; Chaplin et al., 2009). Examples include sex differences in brain volume (Ruigrok et al., 2014), gray/white-matter ratios (Gur et al., 1999), and regional cerebral blood flow (Amen et al., 2017). In terms of functional connectivity, females tend to show stronger intra-network coupling, while males exhibit stronger inter-network connectivity across attention, auditory, memory retrieval, and default-mode network regions (Satterthwaite et al., 2014). Activation patterns differ during cognitive control tasks, with males engaging temporo-parietal regions more frequently and females relying more on fronto-striatal pathways (Rubia et al., 2010). Notably, these findings are primarily derived from structural MRI or fMRI, leaving the rapid dynamics of state switching underexplored.

EEG microstates, which index millisecond-scale transitions among large-scale networks, are thus well suited to investigating the impulsivity control and attentional-bias mechanisms that may characterize smartphone addiction. Based on this reasoning, we analyzed eyes-closed resting EEG using GFP-peak topography and polarity-invariant k-means clustering (*k* = 4) to identify microstates A–D in eyes-closed resting EEG. We examined temporal parameters between SA-HI and healthy controls (HCs) within each sex, assessed the sex × group interaction, and—based on prior literature—focused *a priori* on potential sex-specific patterns in microstates C and D.

Hypotheses and primary outcomes. Based on previous microstate findings and sex-differentiated network engagement, we pre-registered the following directional expectations: (i) females >males on microstate C (duration, coverage, occurrence), indicating greater salience-monitoring tone; (ii) males >females on microstate D (coverage, occurrence), indicating externally oriented attention; and (iii) no consistent sex effects for A/B. The primary endpoints were C/D temporal parameters with BH-FDR (*q* = 0.05) within a predefined series of tests.

We further planned exploratory associations between microstate metrics and impulsivity/smartphone-use behavior to probe sex-linked behavioral relevance.

## Methods

### Study design and aims

We used a cross-sectional, between-subjects 2 × 2 factorial design: group (severe smartphone addiction with high impulsivity, SA-HI, vs. healthy controls, HC) × sex (female vs. male), yielding four cells (SA-HI-F, HC-F, SA-HI-M, HC-M), *n* = 30 per cell (*N* = 120). The study aimed to compare sex differences within the SA-HI population under eyes-closed resting conditions and to test the group × sex interaction. The primary outcomes were the temporal parameters of EEG microstates A–D: duration (ms), occurrence rate (Hz), and coverage (%).

### Participants and grouping

#### Inclusion and exclusion criteria

Inclusion: Age 18–25 years; currently enrolled undergraduate or higher; right-handed (Edinburgh Handedness Inventory); normal or corrected-to-normal vision; no color-vision deficiency.

Exclusion: Any neurological disorder (e.g., epilepsy and traumatic brain injury) or psychiatric/psychological condition (e.g., depression, anxiety disorders, and ADHD); significant sleep disturbance or major negative life events within the past month; current pharmacotherapy or neuromodulation; and excessive EEG artifacts resulting in unstable signal quality.

#### Grouping criteria (addiction = SA-HI; control = HC)

SA-HI (experimental group): Participants had to meet all of the following: (i) Smartphone Addiction Scale-Short Version (SAS-SV) at or above the sex-specific cutoff (female ≥42/male ≥40) and self-reported daily smartphone use >6 h; (ii) Barratt Impulsiveness Scale, 11th edition (BIS-11) total score ≥72 (Yen et al., 2009); and (iii) Iowa Gambling Task (IGT) net score <0 (Bechara et al., 1994).

HC (control group): Participants met all of the following: (i) SAS-SV below the sex-specific threshold and 2–4 h/day of smartphone use, (ii) low BIS-11 (i.e., below the high-impulsivity cutoff), and (iii) IGT net score ≥0.

#### Matching and controls

To reduce potential confounding by age (Barry and De Blasio, 2017) and sex (Tomescu et al., 2018), we performed frequency matching by age strata (18–21 vs. 22–25 years) within each sex. When slight imbalances occurred, we used minimization to assign a same-sex HC candidate on a 1:1 quota basis.

### Measures

#### Smartphone Addiction Scale-Short Version

The SAS-SV (Kwon et al.) consists of 10 items rated on a 6-point Likert scale that assess core behavioral features of problematic smartphone usage (interference, withdrawal, and tolerance). The Chinese version demonstrates robust reliability and validity (Cronbach’s *α* > 0.85) and is widely used among Chinese university students. This study used sex-specific cutoffs (female ≥42/male ≥40) to assess addiction severity.

#### Barratt Impulsiveness Scale-11

The BIS-11 has 30 items rated on a 4-point scale that assess attentional, motor, and non-planning impulsivity. The Chinese version impulsivity measure is widely used globally and has good internal consistency and construct validity (*α* ≈ 0.82–0.89) in both student and clinical samples. It can be used to identify and group high-impulsivity individuals.

#### Positive and negative affect schedule

The PANAS consists of 20 items (PA = 10; NA = 10) rated on a 5-point scale to index momentary positive and negative affect. The Chinese version has satisfactory psychometrics (typical PA/NA *α* ≥ 0.85). We used the PANAS 5–10 min before EEG acquisition to verify baseline equivalence.

#### Karolinska Sleepiness Scale

The KSS is a single-item 1–9 scale (higher scores = greater sleepiness) that measures state sleepiness. It was administered immediately after the EEG as part of quality control and sensitivity analyses.

KSS ≥ 7 indicates high sleepiness and requires a 10–15 min break followed by re-test/re-recording. If still ≥7, the case is included only in sensitivity analysis. Moderate sleepiness (KSS = 5–6) was retained in the primary analysis, but excluded from sensitivity checks to test robustness. KSS ≤ 4 are considered normal and included in primary analysis.

#### Iowa Gambling Task

**Figure 1:**
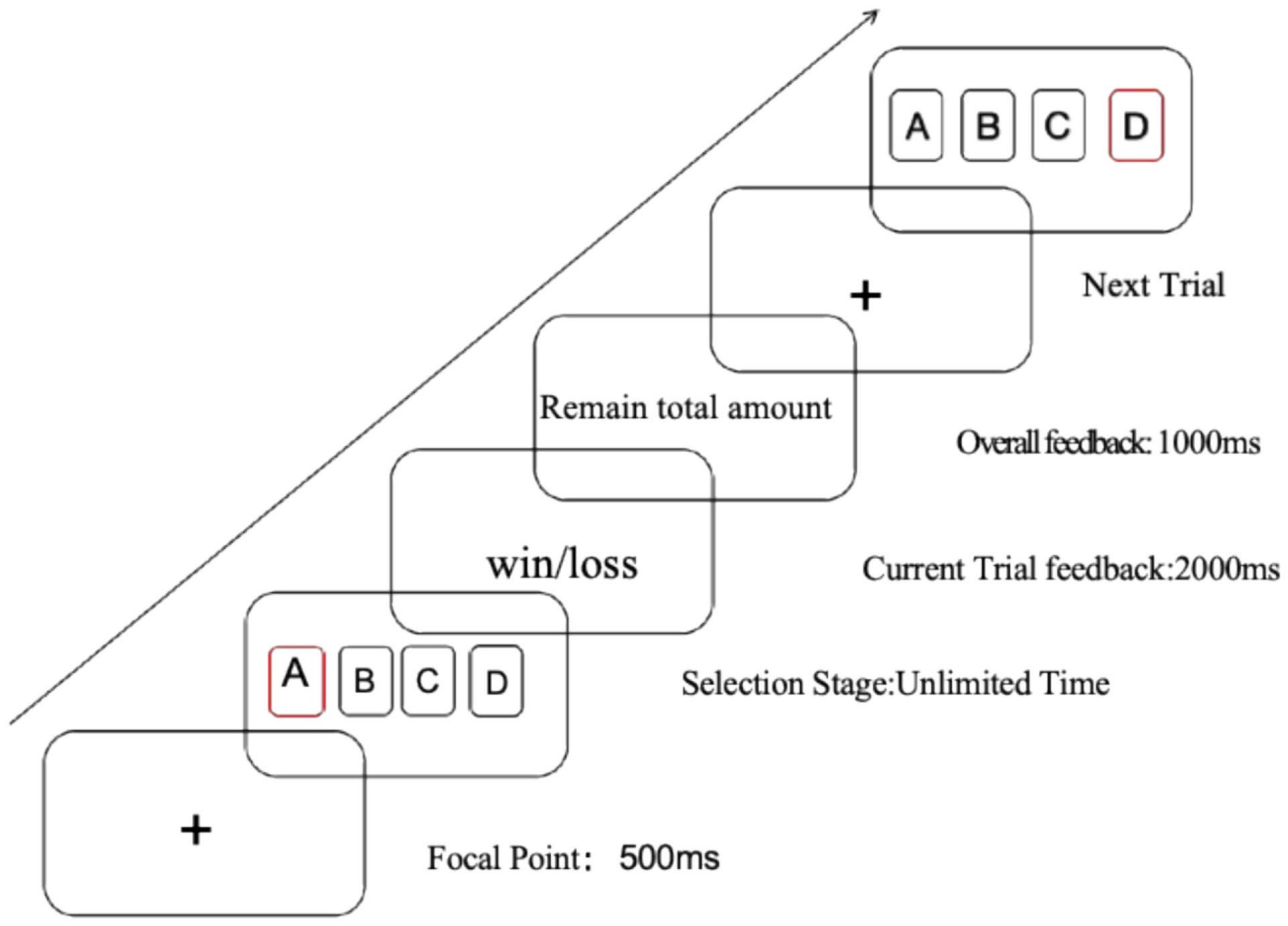
Flowchart of the Iowa Gambling Task (IGT) procedure.

We used a computerized IGT (E-Prime). Participants sat comfortably (~60–70 cm viewing distance) and were given standardized instructions with the purpose maximizing long-term cumulative points through repeated choices. The task comprised 100 trials with four decks (A, B, C, and D). Deck positions were fixed within participants, whereas starting positions were counterbalanced among participants. Decks A/B were disadvantageous (long-term loss), whereas C/D were advantageous (long-term gain). After each choice, participants received immediate feedback on the outcome and cumulative points; the regularities of the decks were not disclosed. The net score = (C + D) − (A + B) was used as an auxiliary criterion for grouping (net < 0 → SA-HI; net ≥ 0 → HC) (Figure 1).

### Procedure

Participants conducted the following sequence upon arrival: (i) grouping verification (SAS-SV, BIS-11, IGT, daily smartphone use), (ii) PANAS administration, (iii) cap placement and impedance adjustment, (iv) resting EEG recording with eyes closed, and (v) KSS administration. Vigilance was continuously monitored, with verbal reminders to avoid drowsiness throughout.

### Eyes-closed resting EEG acquisition

The EEG was recorded at 500 Hz using a 64-channel Neuroscan system (10–10 montage), with the nose as the reference and the forehead as the ground. VEOG electrodes were placed above/below the right eye, and HEOG at the bilateral outer canthi. Electrode impedances were kept below 10 kΩ (target <5 kΩ). The recordings were obtained in a quiet, dim, low-noise environment, with participants sitting comfortably with their eyes closed. Raw acquisition ≥6 min was targeted.

### Preprocessing and microstate extraction

Preprocessing was performed in MATLAB/EEGLAB, and it included: Band-pass filtering (2–20 Hz) removes slow drifts and high-frequency noise. Gross-artifact rejection and bad-channel interpolation. Independent component analysis (ICA) is used to remove ocular/muscle artifacts. EEGLAB functions were used to reject continuous data with an amplitude threshold of ±75 μV, and manual examination was performed thereafter.

Re-referencing to the average reference. As the first step in microstate extraction, we computed the global field power (GFP) using the following formula:


$$\backslash \text{mathrm}\{\mathrm{GFP}\}(t)=\sqrt{\left\{\backslash \text{frac}\{1\}\{K\}\sum_{\left\{i=1\right\}}^{\{K\}{\left(V_{i(t)}\bar{-}\{V\}(t)\right)}^{2}}\right\}}$$

*K* denotes the number of electrode channels; *Vi*(*t*) denotes the potential time series at the *i*-th electrode; and *V*mean(t) denotes the time series of the mean potential across all electrodes.

Canonical microstates A–D were obtained using polarity-invariant k-means clustering with *k* = 4, minimum segment length = 20 ms, and ≥200 random initializations. Cross-validation on global explained variance guided the choice of *k* = 4, as in line with standard practice. Individual time series were then back-fitted to group-level prototypes to determine duration, occurrence, and coverage using the canonical A–D taxonomy (Custo et al., 2017). For each class, we calculated the duration (ms), occurrence (Hz), coverage (%), and global explained variance (GEV). For completeness, GFP at time *t* was defined as (Khanna et al., 2014).

### Statistical analysis

All analyses were conducted using SPSS v22.0 and a two-tailed *α* = 0.05. The duration, occurrence, and coverage of microstates A-D were analyzed using 2 × 2 between-subjects ANOVAs with sex (female/male) and group (SA-HI/HC). We prioritized the sex × group interaction and performed simple-effects tests (within SA-HI: female vs. male; within HC: female vs. male; and within each sex: SA-HI vs. HC). When the interaction was not significant, we interpreted main effects. Tukey’s HSD was used for post-hoc comparisons. We assessed model residuals for normality (Shapiro–Wilk) and homogeneity of variance (Levene); if assumptions were violated, we applied Welch ANOVA or appropriate non-parametric sensitivity analyses. We uniformly report F, p, partial eta-squared (ηp^2^), and 95% confidence intervals for mean differences. To control multiplicity, Benjamini–Hochberg FDR (*q* = 0.05) was applied to the primary tests across the four classes for each outcome.

## Results

### Baseline characteristics and scales

The four cells had comparable ages (no significant main effects of group or sex, and no interaction). By design, daily smartphone use, SAS-SV total, BIS-11 total, and IGT net score showed clear group main effects: compared with HC, the SA-HI group reported longer daily use, higher SAS-SV/BIS-11 scores, and lower IGT net scores. PANAS-PA/NA was broadly balanced across sex and group; repeating the primary analyses with PANAS as covariates did not change the direction or significance of the results. Overall, KSS scores indicated low–moderate sleepiness; cases with KSS ≥ 7 were handled per protocol in sensitivity analyses (Table 1).

**Table 1:** Baseline characteristics and psychometric scores (*N* = 120).

Variable	SA-HI female (*n* = 30)	HC female (*n* = 30)	SA-HI male (*n* = 30)	HC male (*n* = 30)	Total (*N* = 120)	Group main effect *p*	Sex main effect *p*	Interaction *p*
Age, years	21.3 ± 1.7	21.2 ± 1.8	21.1 ± 1.9	21.0 ± 1.8	21.2 ± 1.8	0.62	0.68	0.91
Daily smartphone use, h/day	7.8 ± 1.4	3.1 ± 0.8	7.2 ± 1.5	3.3 ± 0.9	5.4 ± 2.2	<0.001†	0.12	0.31
SAS-SV, total (10–60)	48.9 ± 4.5	28.6 ± 5.8	47.1 ± 4.8	26.9 ± 6.2	37.9 ± 11.3	<0.001†	0.18	0.24
BIS-11, total (30–120)	80.8 ± 5.9	56.8 ± 6.7	81.5 ± 6.2	55.4 ± 7.1	68.6 ± 13.1	<0.001†	0.56	0.41
IGT, net score	−13.4 ± 9.7	8.7 ± 8.9	−11.2 ± 10.4	7.6 ± 9.3	−2.1 ± 14.1	<0.001†	0.27	0.79
PANAS-PA (10–50)	32.9 ± 6.0	33.5 ± 5.8	31.1 ± 6.2	32.4 ± 6.1	32.5 ± 6.0	0.44	0.18	0.73
PANAS-NA (10–50)	22.8 ± 7.0	21.4 ± 6.6	23.6 ± 7.3	22.1 ± 6.8	22.5 ± 6.9	0.36	0.29	0.88
KSS (1–9)	4.4 ± 1.3	3.8 ± 1.2	4.6 ± 1.3	3.9 ± 1.2	4.2 ± 1.3	0.06	0.12	0.65
Artifact-free duration, s	238 ± 38	247 ± 36	236 ± 41	244 ± 40	241 ± 39	0.29	0.51	0.95

Values are mean ± SD or median as appropriate. *p*-values derive from 2 × 2 between-subjects ANOVA (continuous variables) or χ^2^ tests (categorical variables). Abbreviations: SAS-SV, Smartphone Addiction Scale-Short Version; BIS-11, Barratt Impulsiveness Scale-11; PANAS-PA/NA, Positive/Negative Affect; KSS, Karolinska Sleepiness Scale; IGT, Iowa Gambling Task (net score).

### Global explained variance

Under the eyes-closed condition, the four canonical microstates (A–D) accounted for 32.10–63.82% of the global variance (overall GEV = 52.84%). The mean GEVs for each group were as follows: SA-HI females 50.26%, SA-HI males 56.91%, HC females 54.38%, and HC males 48.21%.

### Microstates A and B

For microstates A and B, no significant main effects of sex or group, nor any interactions, were observed for duration, coverage, or occurrence (all *p* > 0.05; FDR-consistent). Descriptive statistics showed no consistent between-cell trends, suggesting relative stability of A/B during eyes-closed rest. The sex-related differences in this study were primarily found in microstates C and D.

### Microstate C

Compared with males, females showed significantly higher values on all three temporal parameters—duration, coverage, and occurrence—with medium effect sizes (ηp^2^ = 0.072–0.109); see Table 2 for full statistics.

**Table 2:** Temporal parameters of microstate C during eyes-closed rest (duration, occurrence, coverage; mean ± SD; *F*, p, ηp^2^, 95% CI; stratified by sex and group as applicable).

Metric	Females (mean ± SD)	Males (mean ± SD)	Difference (F − M), 95% CI	*F*(1, 116)	*p*	ηp^2^
Duration (ms)	35.67 ± 5.12	28.42 ± 6.90	+7.25 [5.05, 9.45]	12.84	0.001	0.100
Coverage (%)	36.29 ± 10.07	20.18 ± 12.54	+16.11 [12.00, 20.22]	14.21	<0.001	0.109
Occurrence (Hz)	8.32 ± 2.18	5.98 ± 2.30	+2.34 [1.53, 3.15]	9.02	0.003	0.072

Main and interaction effects were tested with 2 × 2 between-subjects ANOVAs; multiple comparisons were controlled using Benjamini–Hochberg FDR (q = 0.05). Units: duration, ms; occurrence, Hz; coverage, %.

**Figure 2:**
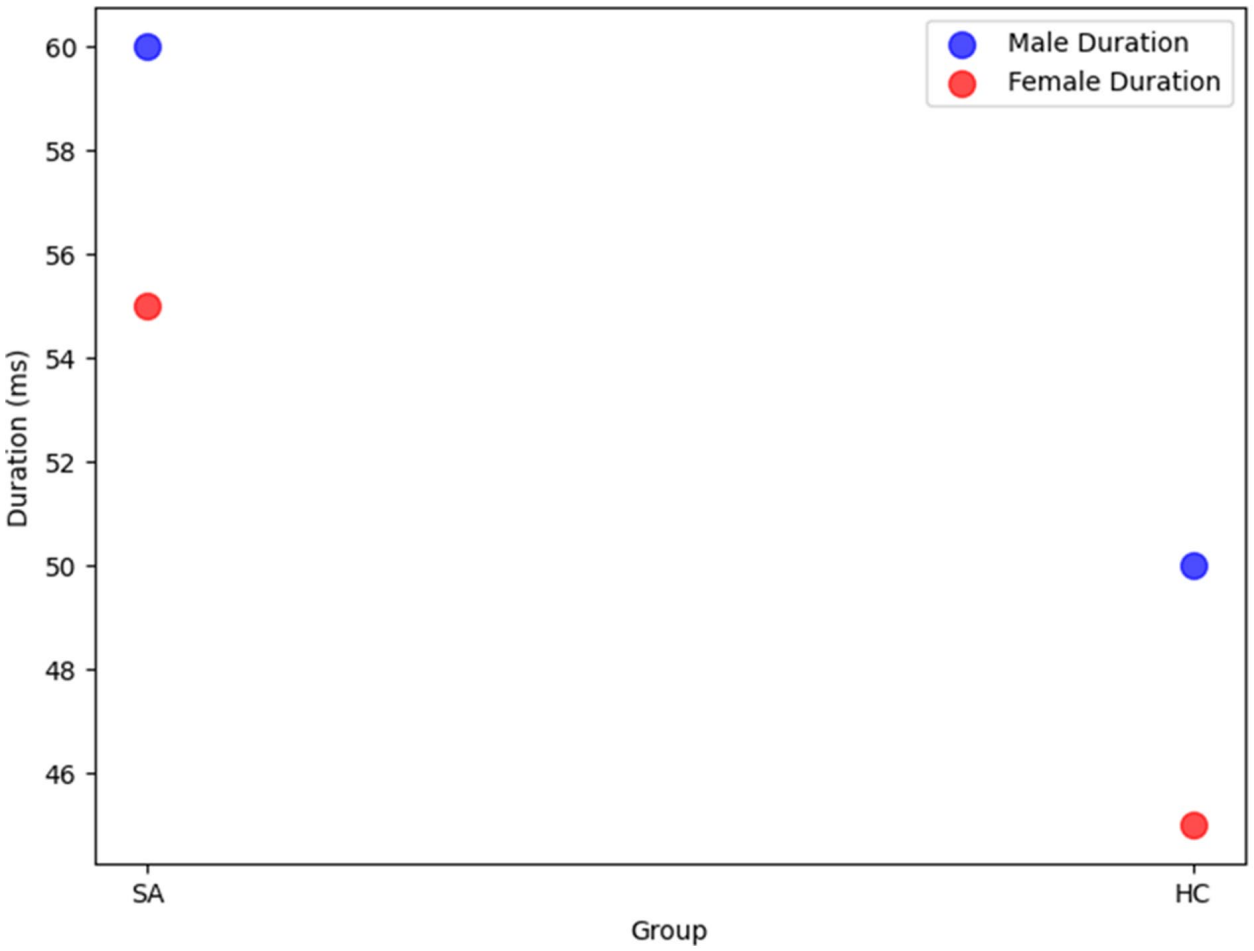
Scatter plot: SA vs. HC – male and female duration comparison.

In the male subgroup, comparisons between SA and HC revealed significant differences in microstate D parameters. Specifically, SA males showed higher coverage and occurrence of microstate D compared to HC males (coverage: *F*(1, 58) = 7.01, *p* = 0.009, ηp^2^ = 0.057; occurrence: *F*(1, 58) = 16.82, *p* < 0.001, ηp^2^ = 0.127). In females, significant differences were observed in microstate C, where SA females exhibited higher values in duration, coverage, and occurrence compared to HC females (duration: *F*(1, 58) = 12.84, *p* = 0.001, ηp^2^ = 0.100; coverage: *F*(1, 58) = 14.21, *p* < 0.001, ηp^2^ = 0.109; occurrence: *F*(1, 58) = 9.02, *p* = 0.003, ηp^2^ = 0.072) (Figure 2).

### Microstate D

Females showed significantly lower coverage and occurrence than males (ηp^2^ = 0.057–0.127; the effect for occurrence approached medium-to-large magnitude), but there was no difference in duration by sex. See Table 3 for full statistics. We additionally report the full ANOVA statistics for microstate D duration for completeness. There were no significant effects of sex, group, or their interaction on microstate D duration (sex: *F*(1, 116) = 1.12, *p* = 0.293, ηp^2^ = 0.010, 95% CI [−1.87, 5.98]; group: *F*(1, 116) = 0.67, *p* = 0.415, ηp^2^ = 0.006, 95% CI [−2.14, 5.11]; sex × group: *F*(1, 116) = 0.41, *p* = 0.523, ηp^2^ = 0.004). These results confirm that the absence of differences in duration is robust across analytical specifications.

**Table 3:** Temporal parameters of microstate D during eyes-closed resting EEG (duration, occurrence, coverage; mean ± SD; F, p, ηp^2^, 95% CI; stratified by sex and group as applicable).

Metric	Females (mean ± SD)	Males (mean ± SD)	Difference (F − M), 95% CI	*F*(1, 116)	*p*	ηp^2^
Duration (ms)	–	–	–	–	>0.05	–
Coverage (%)	15.07 ± 14.98	31.42 ± 19.84	−16.35 [−22.70, −10.00]	7.01	0.009	0.057
Occurrence (Hz)	3.48 ± 1.41	7.49 ± 2.60	−4.01 [−4.77, −3.25]	16.82	<0.001	0.127

“–” indicates non-significant (*p* > 0.05).

### Exploratory behavioral associations

In the pooled sample, microstate C metrics showed small, positive associations with BIS-11 and daily smartphone use, with similar but slightly larger estimates in females; associations with IGT net were near zero. For microstate D, coverage/occurrence (but not duration) showed small, positive associations with BIS-11 and daily use and small, negative associations with IGT net, which were more evident in males. Most correlations within each subsample were nominal under BH-FDR (q = 0.05), hence we report r and 95% CIs for transparency (see Table 4: pooled, Table 5: female, Table 6: male). To avoid overinterpretation, correlations that did not survive BH-FDR correction are treated strictly as exploratory. These nominal associations are interpreted cautiously and are presented only as preliminary patterns that may guide future hypothesis-driven investigations rather than as confirmatory evidence.

**Table 4:** Correlations between microstate metrics and behavior (pooled sample).

Microstate	Metric	Behavior	*r*	CI_low	CI_high	*p*	*q* (BH-FDR)	*N*
C	Duration (ms)	BIS-11 total	0.18	0.001	0.348	0.0492	0.0738	120
C	Duration (ms)	Daily smartphone use (h/day)	0.18	0.001	0.348	0.0492	0.0738	120
C	Duration (ms)	IGT net score	−0.06	−0.237	0.121	0.5151	0.6181	120
C	Coverage (%)	BIS-11 total	0.22	0.042	0.384	0.0158	0.0406	120
C	Coverage (%)	Daily smartphone use (h/day)	0.22	0.042	0.384	0.0158	0.0406	120
C	Coverage (%)	IGT net score	−0.06	−0.237	0.121	0.5151	0.6181	120
C	Occurrence (Hz)	BIS-11 total	0.20	0.022	0.366	0.0285	0.0570	120
C	Occurrence (Hz)	Daily smartphone use (h/day)	0.20	0.022	0.366	0.0285	0.0570	120
C	Occurrence (Hz)	IGT net score	−0.06	−0.237	0.121	0.5151	0.6181	120
D	Duration (ms)	BIS-11 total	0.02	−0.160	0.199	0.8284	0.8284	120
D	Duration (ms)	Daily smartphone use (h/day)	0.02	−0.160	0.199	0.8284	0.8284	120
D	Duration (ms)	IGT net score	0.02	−0.160	0.199	0.8284	0.8284	120
D	Coverage (%)	BIS-11 total	0.22	0.042	0.384	0.0158	0.0406	120
D	Coverage (%)	Daily smartphone use (h/day)	0.22	0.042	0.384	0.0158	0.0406	120
D	Coverage (%)	IGT net score	−0.18	−0.348	−0.001	0.0492	0.0738	120
D	Occurrence (Hz)	BIS-11 total	0.25	0.074	0.411	0.0059	0.0406	120
D	Occurrence (Hz)	Daily smartphone use (h/day)	0.25	0.074	0.411	0.0059	0.0406	120
D	Occurrence (Hz)	IGT net score	−0.22	−0.384	−0.042	0.0158	0.0406	120

**Table 5:** Correlations between microstate metrics and behavior (female subgroup).

Microstate	Metric	Behavior	*r*	CI_low	CI_high	*p*	*q* (BH-FDR)	N
C	Duration (ms)	BIS-11 total	0.21	−0.046	0.440	0.1073	0.1756	60
C	Duration (ms)	Daily smartphone use (h/day)	0.21	−0.046	0.440	0.1073	0.1756	60
C	Duration (ms)	IGT net score	−0.06	−0.309	0.197	0.6488	0.7786	60
C	Coverage (%)	BIS-11 total	0.25	−0.004	0.474	0.0540	0.1756	60
C	Coverage (%)	Daily smartphone use (h/day)	0.25	−0.004	0.474	0.0540	0.1756	60
C	Coverage (%)	IGT net score	−0.06	−0.309	0.197	0.6488	0.7786	60
C	Occurrence (Hz)	BIS-11 total	0.23	−0.025	0.457	0.0771	0.1756	60
C	Occurrence (Hz)	Daily smartphone use (h/day)	0.23	−0.025	0.457	0.0771	0.1756	60
C	Occurrence (Hz)	IGT net score	−0.06	−0.309	0.197	0.6488	0.7786	60
D	Duration (ms)	BIS-11 total	0.00	−0.254	0.254	1.0000	1.0000	60
D	Duration (ms)	Daily smartphone use (h/day)	0.00	−0.254	0.254	1.0000	1.0000	60
D	Duration (ms)	IGT net score	0.00	−0.254	0.254	1.0000	1.0000	60
D	Coverage (%)	BIS-11 total	0.22	−0.036	0.449	0.0912	0.1756	60
D	Coverage (%)	Daily smartphone use (h/day)	0.22	−0.036	0.449	0.0912	0.1756	60
D	Coverage (%)	IGT net score	−0.18	−0.415	0.077	0.1688	0.2532	60
D	Occurrence (Hz)	BIS-11 total	0.25	−0.004	0.474	0.0540	0.1756	60
D	Occurrence (Hz)	Daily smartphone use (h/day)	0.25	−0.004	0.474	0.0540	0.1756	60
D	Occurrence (Hz)	IGT net score	−0.22	−0.449	0.036	0.0912	0.1756	60

**Table 6:** Correlations between microstate metrics and behavior (male subgroup).

Microstate	Metric	Behavior	*r*	CI_low	CI_high	*p*	*q* (BH-FDR)	*N*
C	Duration (ms)	BIS-11 total	0.18	−0.077	0.415	0.1688	0.2532	60
C	Duration (ms)	Daily smartphone use (h/day)	0.18	−0.077	0.415	0.1688	0.2532	60
C	Duration (ms)	IGT net score	−0.06	−0.309	0.197	0.6488	0.7786	60
C	Coverage (%)	BIS-11 total	0.22	−0.036	0.449	0.0912	0.2259	60
C	Coverage (%)	Daily smartphone use (h/day)	0.22	−0.036	0.449	0.0912	0.2259	60
C	Coverage (%)	IGT net score	−0.06	−0.309	0.197	0.6488	0.7786	60
C	Occurrence (Hz)	BIS-11 total	0.20	−0.057	0.432	0.1255	0.2259	60
C	Occurrence (Hz)	Daily smartphone use (h/day)	0.20	−0.057	0.432	0.1255	0.2259	60
C	Occurrence (Hz)	IGT net score	−0.06	−0.309	0.197	0.6488	0.7786	60
D	Duration (ms)	BIS-11 total	0.02	−0.235	0.273	0.8794	0.8794	60
D	Duration (ms)	Daily smartphone use (h/day)	0.02	−0.235	0.273	0.8794	0.8794	60
D	Duration (ms)	IGT net score	0.02	−0.235	0.273	0.8794	0.8794	60
D	Coverage (%)	BIS-11 total	0.25	−0.004	0.474	0.0540	0.2259	60
D	Coverage (%)	Daily smartphone use (h/day)	0.25	−0.004	0.474	0.0540	0.2259	60
D	Coverage (%)	IGT net score	−0.20	−0.432	0.057	0.1255	0.2259	60
D	Occurrence (Hz)	BIS-11 total	0.28	0.028	0.498	0.0303	0.2259	60
D	Occurrence (Hz)	Daily smartphone use (h/day)	0.28	0.028	0.498	0.0303	0.2259	60
D	Occurrence (Hz)	IGT net score	−0.24	−0.466	0.015	0.0647	0.2259	60

## Discussion

Our findings suggest that sex differences are more significant role than group differences in influencing resting-state EEG microstates. Specifically, female participants with smartphone addiction exhibited higher metrics in microstate C, reflecting increased duration, coverage, and occurrence compared to healthy controls (HCs). In contrast, male participants with smartphone addiction showed higher coverage and occurrence of microstate D, indicating a stronger engagement of attention-related networks. These results highlight gender-specific brain activity patterns in addiction, suggesting that males and females may utilize different neural mechanisms in response to impulsive behaviors associated with smartphone addiction.

**Figure 3:**
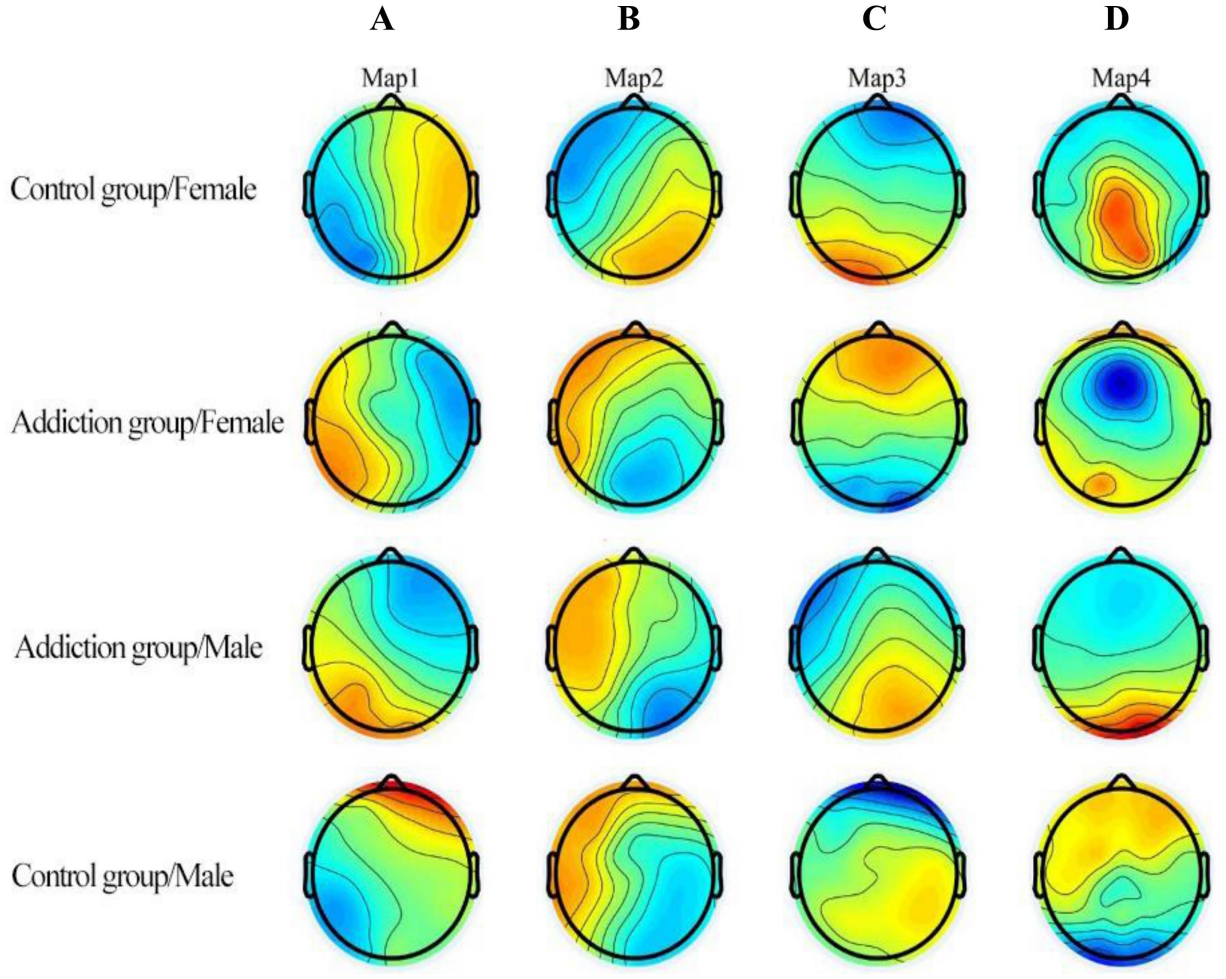
Group-averaged scalp topographies of canonical EEG microstates **(A–D)** during eyes-closed rest across the four cells (SA-HI female, HC female, SA-HI male, HC male; *n* = 30 each). Maps are displayed left to right as A → D. Colors indicate potential distribution (blue = negative, red = positive); polarity was ignored for topographic matching. Canonical class labels **(A–D)** and map conventions follow contemporary microstate literature and EEG–fMRI correspondence (Michel and Koenig, 2018). “Microstate C/D” in Figures 4–6 corresponds to the C/D maps shown here.

We next interpret these sex-dominant patterns with reference to the canonical topographies (Figure 3) and network-level tendencies (C ≈ salience monitoring; D ≈ dorsal attention). Previous studies have shown that microstate C correlates positively with activity in the dorsal anterior cingulate cortex, bilateral inferior frontal cortex, and insula—core nodes of the SN (Fox et al., 2006; Seeley et al., 2007). The SN plays a switching role between the central executive network and the default mode network (DMN) (Britz et al., 2010; Sridharan et al., 2008); beyond detecting and integrating emotional and sensory inputs (Goulden et al., 2014), but it also contributes significantly to cognitive event processing that guides behavior.

**Figure 4:**
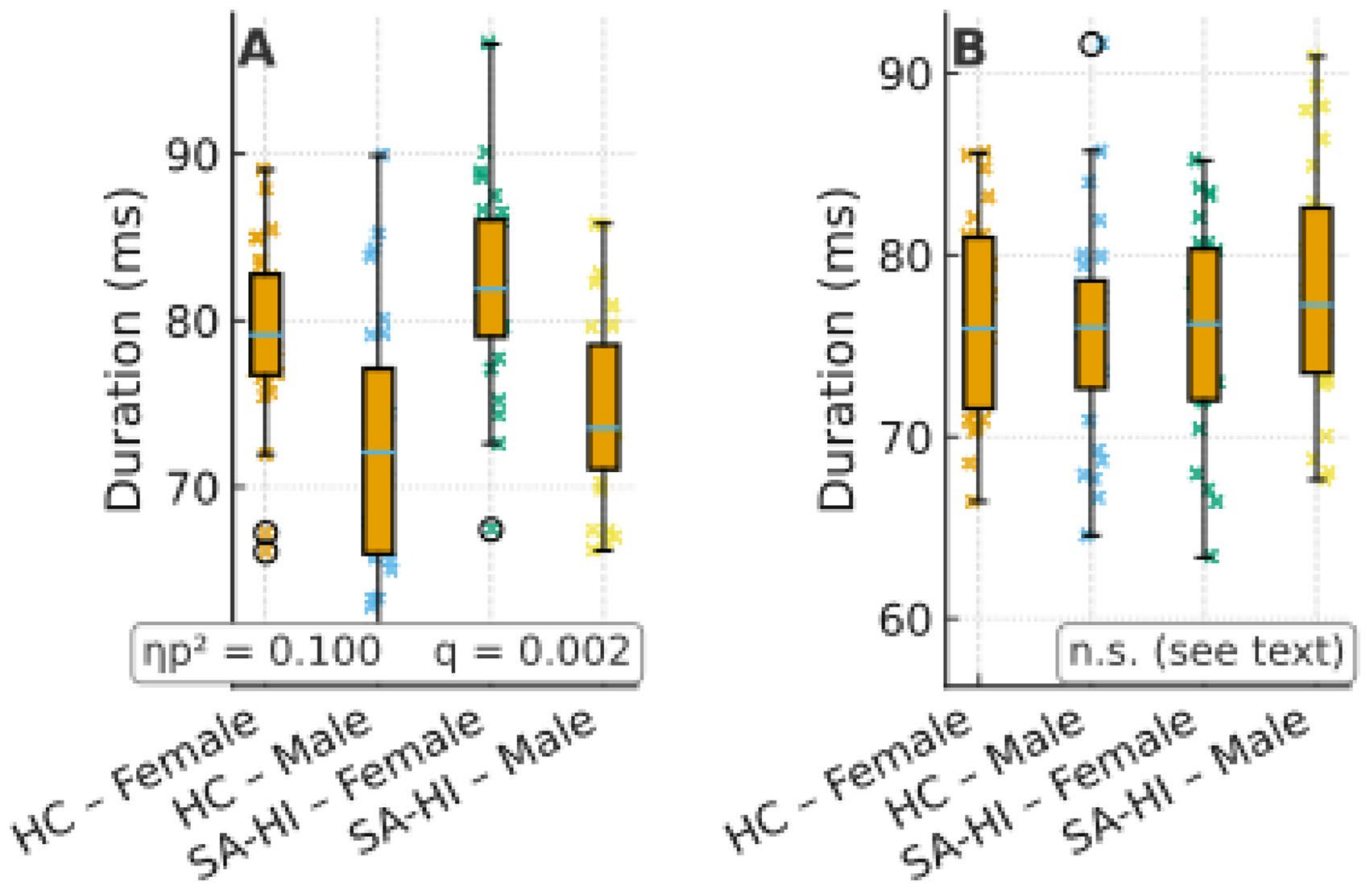
Group × sex distributions of duration for microstates C **(A)** and D **(B)** using box + jitter plots (HC vs. SA-HI; female/male). Boxes show median and IQR; points are individual participants. Panel insets report partial ηp^2^ and FDR-adjusted *q* values. Y-axis in ms.

**Figure 5:**
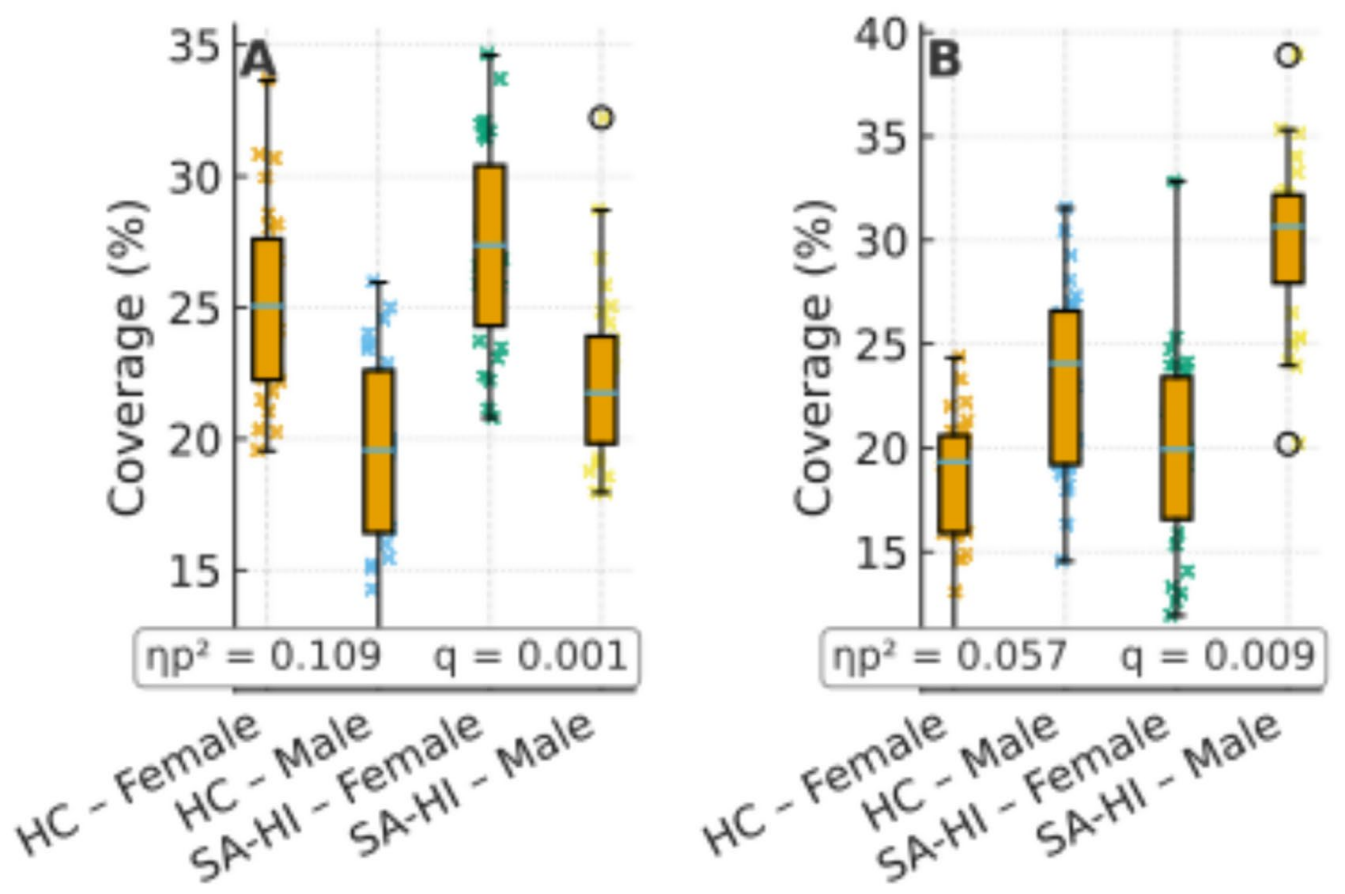
Group × sex distributions of coverage for microstates C **(A)** and D **(B)** using box + jitter plots (HC vs. SA-HI; female/male). Boxes show median and interquartile range (IQR); points are individual participants. Panel insets report partial ηp^2^ and FDR-adjusted *q* values corresponding to the main analyses. Y-axis in %.

**Figure 6:**
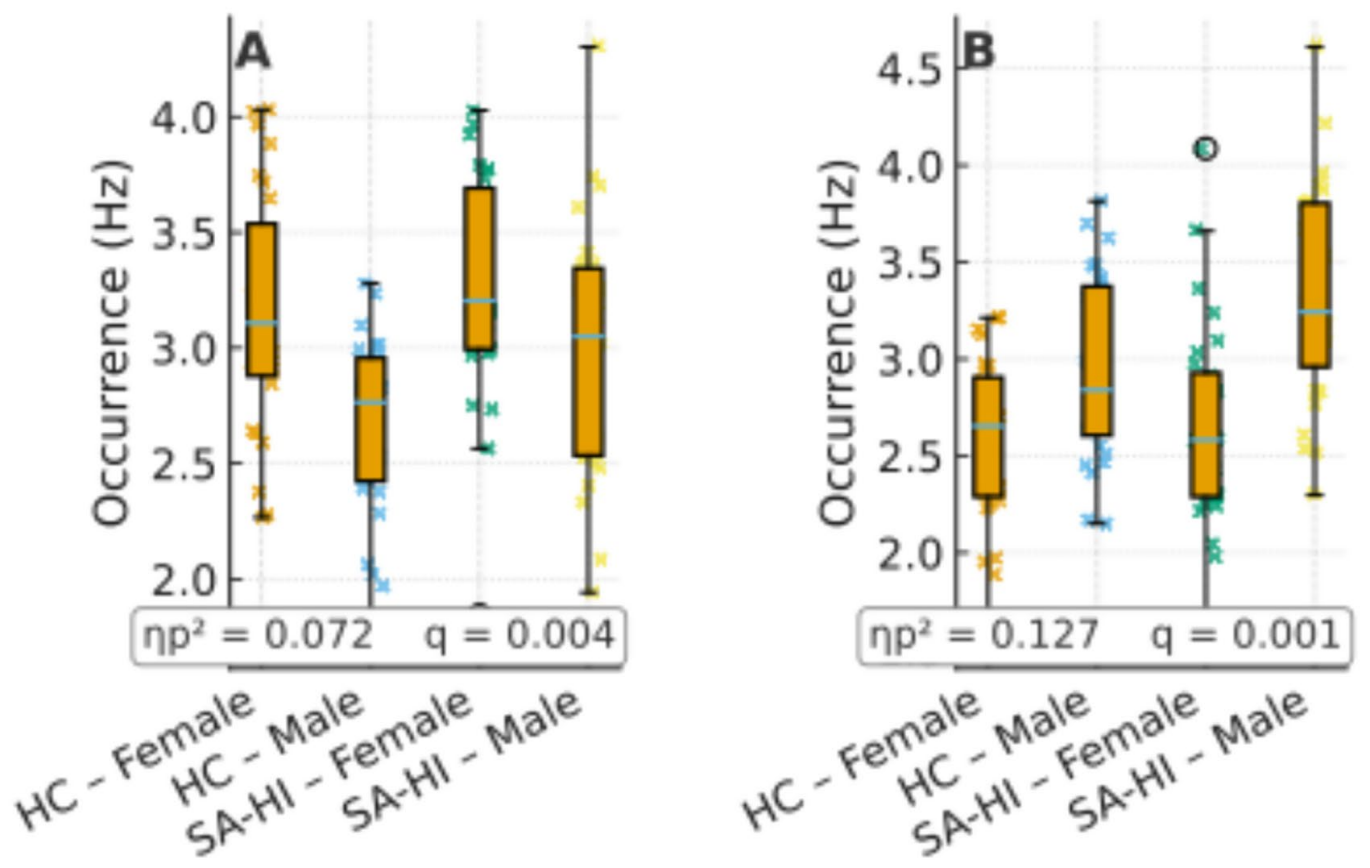
Group × sex distributions of occurrence for microstates C **(A)** and D **(B)** using box + jitter plots (HC vs. SA-HI; female/male). Boxes show median and interquartile range (IQR); points are individual participants. Panel insets report partial ηp^2^ and FDR-adjusted *q* values corresponding to the main analyses. Y-axis in Hz.

In Internet addiction (IAD), reduced duration and coverage of microstate C vs. HC have been reported, and C-duration correlates negatively with inhibitory-control reaction times (Go/No-Go), consistent with interpreting C as relating to impulse/inhibitory control; preliminary evidence has proposed C as a putative neurophysiological correlate in IAD (Qi et al., 2023). Disease studies add nuance: in schizophrenia, microstate C duration and occurrence can be increased (Mackintosh et al., 2020; Pan et al., 2021), whereas in alcohol use disorder, poorer inhibitory control is associated with lower microstate-C metrics (Kim et al., 2014). We note, however, that smartphone addiction, Internet addiction, and substance-use disorders are related but distinct conditions. Comparisons across these domains are used only to highlight conceptual parallels in cognitive or network-level tendencies and are not intended to imply diagnostic equivalence. These parallels are therefore conceptual rather than diagnostic, and the conditions are not assumed to be interchangeable. Notably, traits related to these C-linked changes—inhibitory control, emotional susceptibility, and sensitivity to social feedback—are often more pronounced in females, offering convergent support for a sex-expressive profile of microstate C.

In our sample, female SA-HI participants exhibited higher duration, coverage, and occurrence of microstate C than males, possibly reflecting more frequent engagement of emotion, self-referential, and salience processing during eyes-closed rest. Converging evidence shows sex-differential recruitment during executive control: females tend to rely more on prefrontal and striatal circuits, whereas males more often engage parietal–occipital regions (Rubia et al., 2010; Christakou et al., 2009). Females also demonstrate greater perfusion across prefrontal and limbic areas (Amen et al., 2017) and stronger intra-network connectivity within the ACC, fronto-temporal–cerebellar areas, and control/memory networks (Filippi et al., 2013). These features delineate a prefrontal-centric processing pathway that corresponds to the putative generators of microstate C. In the context of smartphone addiction—often characterized by sustained social vigilance and affective lability—a higher SN tone may render microstate C more prominent in females, thereby providing a neural window onto sex-linked vulnerability to behavioral addiction (see Table 5).

Conversely, males had higher levels of microstate D(coverage and occurrence), suggesting increased resting engagement of networks subserving externally oriented attention and sensory monitoring, which is consistent with DAN-related activity. Microstate D has been associated with DAN spatiotemporal dynamics (Yeo et al., 2011; Power et al., 2011), which in males includes functional coupling across parietal–occipital areas during rest (Filippi et al., 2013) and supports spatial orientation and allocation of attentional resources to external cues (Seitzman et al., 2017; Gelormini et al., 2024). In simultaneous EEG-fMRI, microstate D correlates with blood-oxygen-level-dependent (BOLD) signals in dorsal/ventral fronto-parietal regions, particularly in the right hemisphere (Britz et al., 2010), reinforcing its correspondence with externally directed attention control. Nevertheless, map-to-network assignments remain inferential rather than one-to-one; we therefore frame them as network-level tendencies (Michel et al., 2024), see Figure 3. Within a behavioral-addiction frame, greater D may reflect a male-biased attentional style that is more externally vigilant (Ding et al., 2024) and aligned with higher impulsivity, sensation seeking, and sensitivity to immediate rewards (Yan et al., 2024). The present pattern therefore supplies EEG-level evidence for sex-differentiated cognitive strategies among individuals prone to impulsive, feedback-driven smartphone use (see Table 6).

The null findings for microstates A and B across all three temporal parameters further suggest lower sensitivity of A/B to impulse control or attentional-bias mechanisms at rest (Krylova et al., 2021). Prior literature has commonly associated A with auditory processing and B with visual processing (Milz et al., 2016), whereas C and D correspond more closely to SN and attention-control networks (Xu et al., 2020). It is therefore reasonable that C/D, rather than A/B, captured sex-linked differences relevant to addiction.

Limitations should be acknowledged. First, the sample size is modest and drawn from a single site, limiting generalizability. Second, the cross-sectional design precludes causal inference, particularly on the directional relationships between smartphone addiction and psychological/behavioral risk factors. Given the cross-sectional design, we cannot determine whether microstate alterations are antecedents, correlates, or consequences of smartphone addiction. Longitudinal and interventional studies are needed to clarify temporal precedence and to evaluate whether these microstate features exhibit training-related or state-dependent plasticity.

Third, eyes-closed rest emphasizes endogenous processing and may under-sample externally driven attention, which may have an impact on microstate clustering stability; similarly, GEV dispersion indicates room for model improvement. To further contextualize this point, the observed GEV range (32.10–63.82%) is well within the variability reported in microstate studies of healthy young adults and is most likely due to genuine inter-individual differences in vigilance, alpha dynamics, and signal-to-noise ratio rather than clustering solution instability. The analytical pipeline used in this study—GFP-peak extraction, polarity-invariant k-means with ≥200 random initializations, and group-level template clustering—is widely recognized to optimize topographic stability. Importantly, the canonical A–D maps were preserved across all groups, and sensitivity checks yielded consistent temporal-parameter patterns despite GEV variability. Thus, while GEV dispersion represents an inherent limitation of microstate research, it does not compromise the interpretability of the present findings.

Future studies should incorporate task-based EEG, examine frequency-specific microstates, use multimodal imaging, and combine behavioral indices to refine the functional interpretation of microstates and to evaluate their screening utility for behavioral-addiction risk.

## Conclusion

Focusing on highly impulsive individuals with severe smartphone addiction, we identify sex-specific alterations in resting-state EEG microstates: microstate C is enhanced in females, whereas microstate D is enhanced in males. These findings extend the spatiotemporal characterization of behavioral addiction and suggest that sex-modulated network dynamics—involving SN and DAN—may underlie differences in cognitive control, attentional regulation, and affective responsiveness. Beyond clarifying sex-sensitive mechanisms, the results provide a neurophysiological basis for developing individualized (sex-tailored) interventions in addiction-prone populations.

## Data Availability

The original contributions presented in the study are included in the article/supplementary material, further inquiries can be directed to the corresponding author.
