# Correction: Sex-dominant differences in resting-state EEG microstates among young adults at risk of smartphone addiction

**DOI:** 10.3389/fnhum.2026.1883797

**Published:** 2026-06-09

**Authors:** Jun Chen, Yingying Zeng, Hui Liu, Danfeng Yu, Bo Yang, Qin Liu

**Affiliations:** 1 Department of Cardiopulmonary Rehabilitation, Taihe Hospital, Hubei University of Medicine, Shiyan, Hubei, China; 2 Department of Cardiology, Taihe Hospital, Hubei University of Medicine, Shiyan, Hubei, China; 3 Department of General Practice, Taihe Hospital, Hubei University of Medicine, Shiyan, Hubei, China; 4 Department of Neurocritical Care, Taihe Hospital, Hubei University of Medicine, Shiyan, Hubei, China; 5 Department of Radiology, Taihe Hospital, Hubei University of Medicine, Shiyan, Hubei, China

**Keywords:** EEG microstates, impulsivity, resting state, sex differences, smartphone addiction

**Affiliation** 2 Department of Cardiology, Taihe Hospital, Hubei University of Medicine, Shiyan, Hubei, China was omitted for author Yingying Zeng. This affiliation has now been replaced for author Yingying Zeng.

**Affiliation** 3 Department of General Practice, Taihe Hospital, Hubei University of Medicine, Shiyan, Hubei, China was omitted for author Hui Liu. This affiliation has now been replaced for author Hui Liu.

**Affiliation** 4 Department of Neurocritical Care, Taihe Hospital, Hubei University of Medicine, Shiyan, Hubei, China was omitted for author Danfeng Yu. This affiliation has now been replaced for author Danfeng Yu.

**Affiliation** 5 Department of Radiology, Taihe Hospital, Hubei University of Medicine, Shiyan, Hubei, China was omitted for author Bo Yang. This affiliation has now been replaced for author Bo Yang.

The original version of this article has been updated.

